# Single stage laparoscopic cholecystectomy with intraoperative endoscopic retrograde cholangiopancreatography for cholecysto-choledocholithiasis. Lesson learnt from the COVID-19 pandemic

**DOI:** 10.3389/fsurg.2024.1398854

**Published:** 2024-06-18

**Authors:** Martino Gerosa, Angelo Guttadauro, Domenico Francesco Stillittano, Richard Sassun, Annaclara Sileo, Barbara Vignati, Emanuele Di Fratta, Dario Maggioni, Giulio Mari

**Affiliations:** ^1^ Laparoscopic and Oncological General Surgery Department, Desio Hospital, ASST Brianza, Desio, Italy; ^2^ Department of Medicine and Surgery, School of Medicine and Surgery, University of Milano Bicocca, Milan, Italy; ^3^ Endoscopy Unit, Desio Hospital, ASST Brianza, Desio, Italy; ^4^ General Surgery Residency, University of Milan, Milan, Italy

**Keywords:** cholecysto-choledocholithiasis, laparoscopic cholecystectomy, endoscopic retrograde cholangiopancreatography, laparoendoscopic rendezvous, laparoscopic common bile duct exploration, COVID-19 pandemic

## Abstract

**Introduction:**

Choledocholithiasis, a common complication of gallstone disease, poses significant risks including cholangitis and pancreatitis. Various treatment approaches exist, including single-stage and two-stage techniques, with recent literature suggesting advantages of the single-stage approach in terms of outcomes and cost-effectiveness. This study evaluates the feasibility, efficacy, and safety of single-stage laparoscopic cholecystectomy combined with intraoperative endoscopic retrograde cholangiopancreatography (LC + iERCP) compared to the previously adopted two-stage approach.

**Methods:**

A retrospective analysis was conducted on patients undergoing single-stage LC + iERCP for cholecysto-choledocholithiasis during the COVID-19 pandemic (2020–2022). Data on demographics, preoperative assessments, intraoperative parameters, and postoperative outcomes were collected and compared with an historical control group undergoing the two-stage approach (LC + preopERCP). Hospitalization costs were also compared between the two groups.

**Results:**

A total of 190 patients were included, with 105 undergoing single-stage LC + iERCP. The single-stage approach demonstrated successful completion without cystic duct cannulation, with no conversions to open surgery. Operative time was comparable to the two-stage approach, while hospital stay, and costs were significantly lower in the single-stage group. Complication rates were similar between the groups.

**Conclusions:**

Single-stage LC + iERCP appears to be a feasible, effective, and safe approach for treating cholecysto-choledocholithiasis, offering potential benefits in terms of reduced hospital stay, OR occupation time, and costs compared to the two-stage approach. Integration of this approach into clinical practice warrants consideration, unless there are logistical challenges that cannot be overcome or lack of endoscopic expertise also for treating challenging urgent cases.

## Introduction

Choledocholithiasis is a consequence of gallstones migration from gallbladder to common bile duct (CBD).

Its prevalence is reported to be 10%–20% among patients with symptomatic cholelithiasis (1). Definitive treatment of cholecysto-choledocholithiasis is advocated to prevent further complications such as cholangitis, acute pancreatitis, or persistent CBD obstruction (2–5).

Laparoscopic cholecystectomy (LC) is considered the gold standard treatment for gallbladder stones. Endoscopic retrograde cholangiopancreatography (ERCP) is the standard procedure for removing CBD stones.

“Single stage” or “two stage” techniques are possible treatment options for cholecysto-choledocholithiasis.

The “Single stage” techniques comprehends: LC and intraoperative ERCP (LC + iERCP); laparoendoscopic rendezvous (LC + LERV); LC with laparoscopic common bile duct exploration (LC + LCBDE).

“Two stage” approach consists in either LC followed by postoperative ERCP (LC + postopERCP) or LC preceded by preoperative ERCP (LC + preopERCP).

Recent papers suggest that the single stage approach is superior due to lower complication rates, shorter hospital stays and lower costs (2, 6).

However, logistical challenges and the need for specialized expertise available often led the two-stage approach in the daily clinical practice.

During the COVID-19 pandemic, efforts to minimize hospital admissions and intra-hospital contamination paved the way to a wider use of the single stage approach. In our department as well the treatment option moved for all patients with cholecysto-choledocholithiasis to single stage laparoscopic cholecystectomy combined with iERCP.

Precisely because already tested in the pre-covid era, this approach was promptly implemented to deal with the problems related to the pandemic.

As a lesson learnt, when the pandemic was over the single stage approach was retained because of its apparent effectiveness. However, a rigorous analysis of the benefits brought about had not yet been carried out in terms of operating times, costs and hospital stay.

In this retrospective study we analyzed a series of LC + iERCP procedures performed for cholecysto-choledocholitiasis in our department during the COVID-19 pandemic from April 2020 to October 2022 matched with a group of consecutive patients treated with LC plus preoperative ERCP (LC + preopERCP) over a period from February 2018 to January 2020.

This study aimed to evaluate whether the single stage LC + iERCP compared to LC + preopERCP could improve operating times, costs and hospital stay being a feasible, effective, and safe procedure for treating cholecysto-choledocholitiasis.

## Materials and methods

We conducted a retrospective analysis of all consecutive patients who underwent either elective or urgent single stage LC + iERCP for cholecysto-choledocholitiasis between April 2020 and October 2022.

In patients with clinical or biochemical signs of cholecysto-choledocholithiasis the diagnosis was confirmed through ultrasound, computed tomography scan, or magnetic resonance cholangiopancreatography (MRCP). Endoscopic ultrasound was performed in patients unsuitable for MRCP.

The data collected included demographic informations (age, sex, BMI), preoperative assessments (comorbidities, anticoagulation/antiplatelet therapy intake, ASA score, laboratory tests, preoperative diagnosis, Sars Cov 2 nasopharyngeal swab results), intraoperative parameters (type of intervention, operative time, conversion rate, CBD clearance, intraoperative complications), and postoperative outcomes [length of hospital stay, complication according to the Clavien-Dindo classification (7), mortality, and COVID-19 related symptoms at hospital discharge and at 15 days follow up].

These data were compared to a group of consecutive patients treated with LC plus preoperative ERCP (LC + preopERCP) over a period from February 2018 to January 2020.

Hospitalization costs, expressed as state reimbursement, were calculated per patient treated with either the single stage or the two-stage approach.

The two interventions were offered exclusively during different time periods, with the two stage cholecystectomy performed between February 2018 to January 2020, and the one stage cholecystectomy between April 2020 and October 2022. This temporal separation minimizes selection bias, as patients did not choose between the two procedures but were assigned based on the period in which they required surgery. Therefore, we did not deemed necessary a propensity score matching.

Categorical variables were reported as frequencies (percent), while continuous variables were reported as mean ± standard deviation (SD) or median with interquartile range according to their distribution. For group comparison, the Chi-Squared test or Fisher's exact test for categorical variables and independent sample *t*-test or Mann–Whitney *U*-test for continuous variables, were used as appropriate.

### Procedure details

#### Two stage technique

Patients diagnosed with cholecysto-choledocholitiasis first underwent ERCP for CBD stone removal and plastic stent placement if needed. ERCP was performed in prone position. Discharge occurred when bilirubin values were in a resolution trend and there were no signs of post-procedural pancreatitis. After 1 month, patients were readmitted for LC and typically discharged after 48 h from surgery if no complications occurred. Laparoscopic cholecystectomy was done in the American position. The CBD plastic stent was then removed 30 days after hospital discharge.

#### Single stage technique

This procedure required the collaboration of a surgical team and an experienced biliary endoscopist.

The cystic duct and artery were dissected from Calot's triangle, with only the artery being ligated and transected. The cystic duct was clipped only on the gallbladder side but not divided. Thus, unlike in LERV, to allow trans cystic guidewire passage if direct endoscopic access to the papilla failed. The gallbladder was then detached from liver's bed.

Following the suspension of pneumoperitoneum, ERCP was performed involving papilla cannulation, sphincterotomy, and cholangiography. Gallstones clearance was achieved through Fogarty angioplasty catheter and/or Dormia's basket. CBD clearance completion was then confirmed through a final cholangiography. Plastic stent may be placed in the CBD if needed to prevent the post-procedural papilla's edema. Intraoperative ERCP was done in supine position. Laparoscopic cholecystectomy was done in the American position.

Subsequently, the pneumoperitoneum was reestablished, the cystic duct was transected, and cholecystectomy was completed. A 7 Ch Jackson-Pratt drainage was left in the Winslow foramen to drain any fluid collection or to address any possible biliary leaks.

## Results

A total of 190 patients were enrolled in the analysis. No difference in the patients' preoperative characteristics were observed at the univariate analysis (Table 1).

**Table 1:** Patients characteristics.

Variable	Single stage (*n* = 105)	Two stage (*n* = 85)	*p*-value
Age, years, median (range)	72 (36–86)	70 (41–78)	0.96
Female gender, *n* (%)	45/105 (42.8)	40/85 (47)	0.65
BMI, median (range)	26 (18–39)	27.1 (19–28.2)	0.78
Preop comorbidities, *n* (%)	54 (51.4)	38 (44.7)	0.09
ASA score, *n* (%)			
I	38 (36.2)	24 (28.2)	0.087
II	42 (40)	40 (47)	0.79
III	25 (23.8)	21 (24.7)	0.64
Urgent procedures	75/105 (71.5)	64/85 (75.3)	0.98
Age, years, median (range)	70 (42–86)	68 (40–77)	0.12
Female gender, *n* (%)	32/75 (42.8)	31/64 (48.4)	0.23
BMI, median (range)	27.2 (20–37)	26.9 (18.3–29.2)	0.18
Preop comorbidities	42/105 (40)	32/85 (37.7)	0.87
Acute cholangitis	25/75 (33.3)	21/64 (32.8)	0.61
Acute cholecistitis	32/75 (42.7)	23/64 (35.9)	0.72
Acute pancreatitis	18/75 (24)	20/64 (31.2)	0.68
ASA score, *n* (%)			
I	25/75 (33.3)	19/64 (29.7)	0.84
II	32/75 (42.7)	25/64 (39)	0.72
III	18/75 (24)	20/64 (31.3)	0.23
Elective procedures	30/105 (28.5)	21/85 (24.7)	0.82
Age, years, median (range)	69 (38–79)	72 (41–81)	0.19
Female gender, *n* (%)	13/30 (43.3)	9/21 (42.8)	0.35
BMI, median (range)	26.8 (22.1–37.4)	27.9 (18.9–29.8)	0.29
Preop comorbidities	12/30 (40)	6/21 (28.6)	0.12
ASA score, *n* (%)			
I	13/30 (43.4)	5/21 (23.8)	0.09
II	10/30 (33.3)	15/21 (71.4)	0.06
III	7/30 (23.3)	1/21 (4.8)	0.08

Among them, 105 patients underwent single stage LC + iERCP.

All patients were Covid 19 free. For Covid 19 positive patients the treatment of choice was ERCP with delayed cholecystectomy within a subsequent admission after recovery from the lung infection.

75/105 patients (71.5%) had an urgent procedure. Of those, 25 patients had cholangitis, 32 patients had cholecystitis and 18 patients had pancreatitis. 30/105 (28.5%) patients had a planned elective procedure for radiologically diagnosed cholecysto-choledocholitiasis. Patients' characteristics for urgent and elective procedure are listed in Table 1.

All procedures were successfully completed without the need for cystic duct cannulation.

Endoscopic sphincterotomy was performed on all patients, and a plastic biliary stent was placed in 84 cases (80%). No conversion to open surgery was required (Table 2).

**Table 2:** Intra- and post-operative parameters.

Variable	Single stage (*n* = 105)	Two stage (*n* = 85)	*p-*value
Operative time, min, median(range)	98 (61–161)	138 (75–195)	0.034
Conversion rate, *n* (%)	0 (0)	1 (1)	0.91
Biliary leak, *n* (%)	1 (1)	0 (0)	0.98
Pancreatitis *n* (%)	8 (7.6)	9 (10)	0.87
Fluid collection *n* (%)	7 (6.5)	6 (7)	0.73

The median operative time was 98 min (range 61–161), and CBD clearance was confirmed by intra operative cholangiography in all patients.

Intraoperative complications, represented by biliary leakage from the hepatic bed occurred only in one patient with acute cholecistitis and was promptly managed by biliary stent placement previously not placed.

The median length of stay was 4 days (range 2–15) with no observed mortality.

The overall complication rate in the single stage group was 42.9% (45/105).

According to the Clavien-Dindo classification (7) grade I and II complications were observed in 34 (32.4%, 10 pneumonia, 7 fluid collection, 6 urinary tract infection, 7 wound infections and 4 pleural effusion) and 8 patients (7.6% 8 pancreatitis), respectively.

Grade III complications occurred in 2 (1.9%) patients. Only one patient, concomitantly treated with Direct Acting Oral Anticoagulation, underwent endoscopic revision and hemostasis for papilla bleeding.

Grade IV complications occurred only in one patient (1%) treated with Low Molecular Weight Heparin for atrial fibrillation and required surgical intervention for hemoperitoneum due to liver subcapsular bleeding (Table 3).

**Table 3:** Postoperative parameters.

Variable	Single stage (*n* = 105)	Two stage (*n* = 85)	*p*-value
Postoperative length of stay, median (range)	4 (2–15)	6 (4–12)	0.042
Clavien Dindo classification, *n* (%)			
I	34 (32.4)	18 (21)	0.053
II	8 (7.6)	9 (10)	0.77
III	2 (1.9)	2 (2)	0.91
IV	1 (1)	0 (0)	0.76
Blood transfusion, *n* (%)	3 (2.9)	4 (5)	0.62
Postoperative mortality, *n* (%)	0 (0)	0 (0)	na
Endoscopic revision, *n* (%)	1 (1)	10 (12)	0.041
Reoperation, *n* (%)	1 (1)	0 (0)	0.8

The control group consisted of 85 patients who underwent the two-stage approach (preop-ERCP followed by LC).

64/85 patients (75.3%) had an urgent procedure. Of those, 21 patients had cholangitis, 23 patients had cholecystitis and 20 patients had pancreatitis. 21/85 (24.7%) patients had a plannes elective procedures for radiologically diagnosed cholecysto-choledocholitiasis. Patients' characteristics for urgent and elective procedure are listed in Table 1.

The median length of stay for the ERCP admissions was 3 days (range 1–7), and the median operative time was 45 min (range 31–74). A plastic biliary stent was placed in 64 patients (75.3%). 10 patients (12%) required a second ERCP for biliary stent dislocation or papilla's bleeding. Postoperative mild pancreatitis occurred in 12 patients (14%) and was treated conservatively.

The median length of stay for the LC admissions was 3 days (range 2–8), with a median operative time of 55 min (range 41–112). In one case conversion to laparotomy was required. The overall complication rate in the two stage group was 34.1% (29/85). Grade I and II complications were observed in 18 (21%, 6 pneumonia, 6 fluid collection and 6 wound infection) and 9 (10%, 9 pancreatitis) patients respectively, while grade III complications occurred in 2 (2%) patients.

64/85 patients (75.3%) had an urgent procedure. In this subgroup of patients, the overall complication rate was 45.3% (29/64) (Table 3).

The median cumulative length of stay for the two staged approach was 6 days (range 4–12) compared to 4 days (range 2–15) for the single stage approach (*p* 0.042).

The median OR occupation time for the two-stage approach was 138 min (range 75–195) compared to 98 min (range 61–161) for the single stage approach (*p* 0.034).

There were no statistically significant differences in the intra/post-operative complication rates between the two groups.

Endoscopic revision was required in 1 patient in the single stage group due to duodenal bleeding (1%) compared to 10 (4 papilla's bleeding and 6 stent displacement) patients in the two-stage group (12%) (*p* 0.041).

There was no statistically significant difference in the overall complication rate in the two groups: single stage group was 42.9% (45/105) in the single stage group vs. 34.1% (29/85) in the two stage group (*p* = 0.71). Focusing on the subgroup of patients who underwent urgent procedure, there was no statistically significant difference in the overall complication rate: 53.3% (40/75) in the single stage group vs. 45.3% (29/64) in the two stage group (*p* = 0.68). There was also no statistically significant difference in the overall complication rate in the elective patients sub-group: 16.5% (5/30) in the single stage group vs. 0% (0/21) in the two stage group.

The hospital costs expressed as state reimbursement per patient were 2889 euros and 4,210 euros for the single stage and the two-stage procedure, respectively.

Patients' characteristics (demographics and preoperative data), intraoperative and postoperative data are detailed in Tables 1–3.

**Figure 1:**
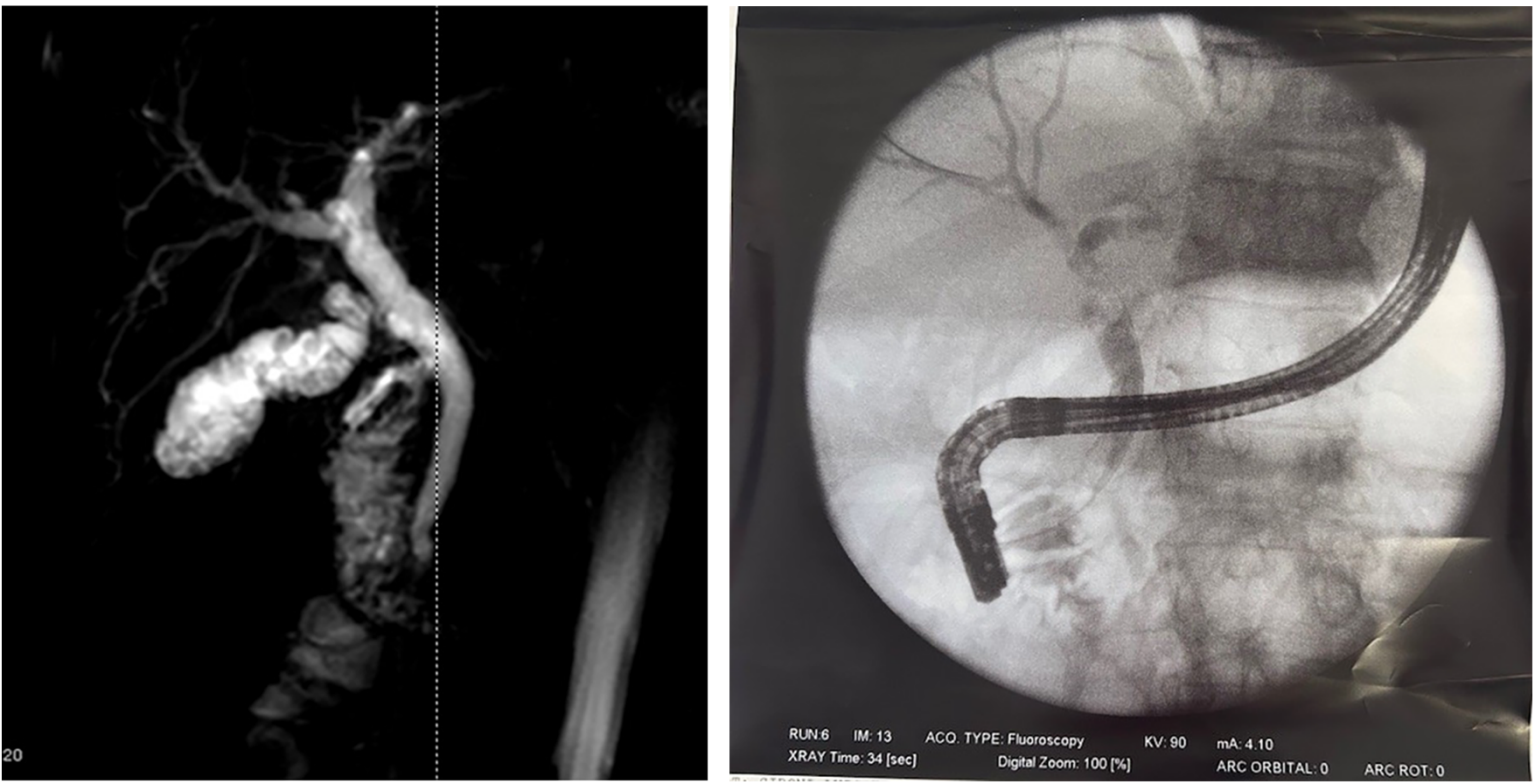
Cholecysto-choledocholithiasis diagnosed at the MRI and Endoscopic retrograde cholangiopancreatography image.

**Figure 2:**
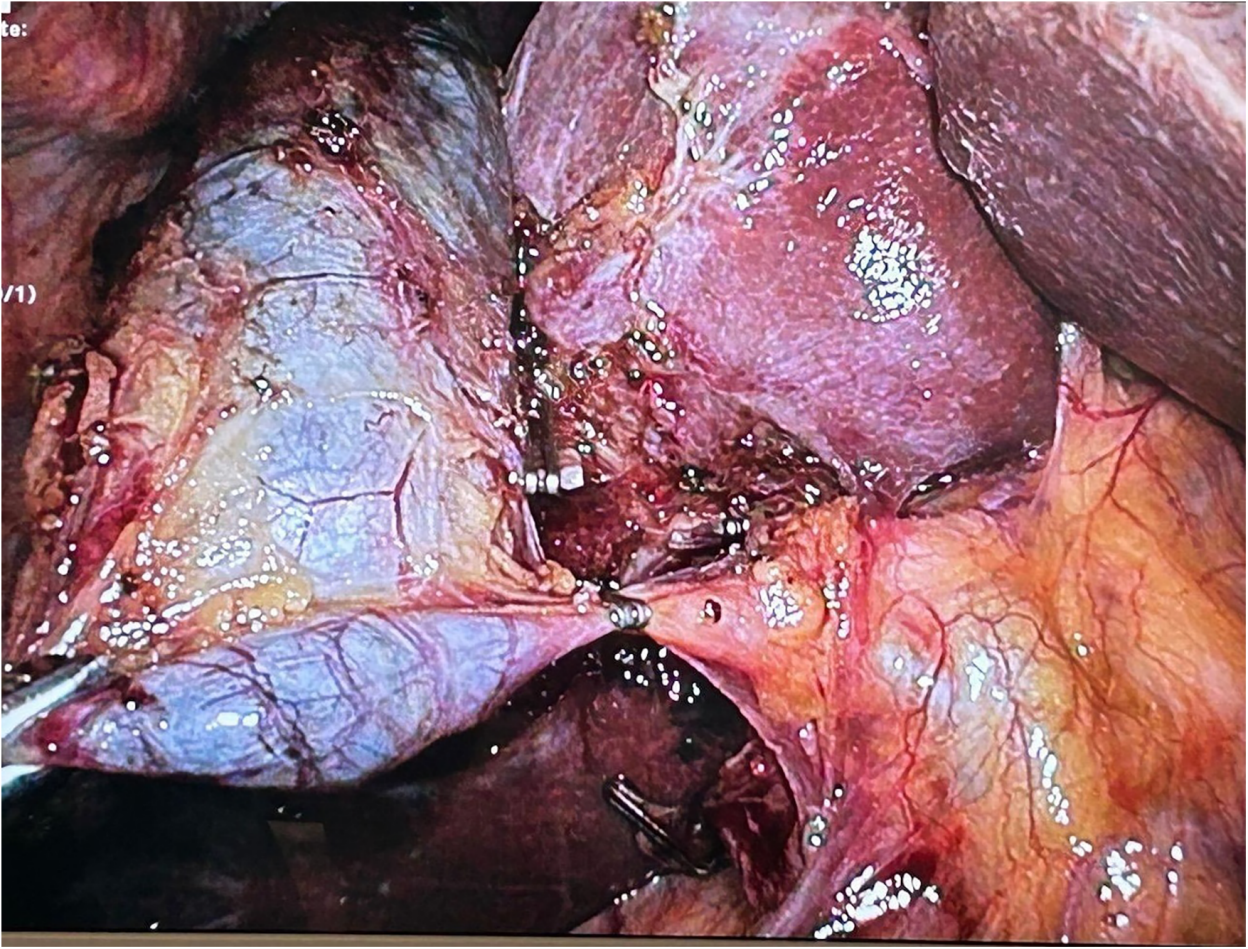
Intraoperative image: the cystic duct and artery were dissected from Calot's triangle, with only the artery being ligated and transected. The cystic duct was clipped only on the gallbladder side but not divided.

Examples of diagnostic, endoscopic and intra-operative images are reported in Figures 1, 2.

## Discussion

During the Covid pandemic, most hospitals were divided into a “clean Covid free” part and a part completely dedicated to the management of Covid patients. The possibility of non-Covid patients becoming infected during hospitalization was real and represented a constant concern. The length of stay of a non-Covid patient certainly represented a risk factor for Covid infection in hospital, as did the need for close re-hospitalizations (8, 9). During Covid pandemic the attempt to concentrate the therapeutic procedures that are routinely spread over multiple admissions within a single hospitalization and in a single intervention was meant to minimize the patient's exposure to Covid contagion.

Compared to preop ERCP + LC, the LC + iERCP approach in fact demonstrates a reduction in hospital stay, OR occupation time, and hospital costs without a significant difference in complication rates.

The need to reduce hospital admissions enforced by the COVID-19 emergency, has become the cornerstone in the single stage treatment of cholecysto-choledocholithiasis.

An unsolved cholecysto-choledocholithiasis could be basically associated to recurrent attack of cholangitis, acute pancreatitis or persistent CBD obstruction thus needing multiple hospital accesses and increasing costs (1, 6, 10).

In our department, after the COVID-19 pandemic, cholecysto-choledocholitiasis was managed by LC and iERCP, except in patients unfit for surgery.

To date the best therapeutic approach for cholecysto-choledocholitiasis remains debated.

A meta-analysis comparing the four techniques previously described suggests single stage procedure's superiority over two stage procedures (11): LCBDE and iERCP are considered safer, more efficient, associated with a shorter hospital stay and with lower costs. However, these procedures are associated with a greater risk for intra operative bleeding. LCBDE reduce the risk for acute pancreatitis but increases the risk of biliary leaks.

A review of 5 randomized controlled trials from China confirmed that the two approaches are comparable in terms of technical success, minor and major morbidity and conversion rate. However, LC + LCBDE is associated with higher biliary leak rate and higher rate of retained stones (10). Despite recent literature favoring single stage procedures, the two-stage approach remains more common in daily clinical practice due to logistical challenges and expertise requirement for the single stage approach.

The benefits of a single stage approach include reduced total hospitalization time, total occupancy of the OR, and overall costs. The data we report appears to be in line with these statements (8, 10).

Concerns surrounding the single stage approach stem from the need of coordination between surgeon and endoscopist. From this point of view the issue seems to be more organizational than linked to the stakeholders' goodwill (2). One thing that could discourage a surgeon from embracing the single stage approach is the possible need to insert a guide wire into the cystic duct if the endoscopist is unable to cannulate the bile duct since such a procedure could be sometimes quite challenging.

However, our series reported a 100% successful endoscopic cannulation without surgical aid, suggesting achievable endoscopic cannulation in expert hands (12).

The complication rate in the two groups did not differ significantly. It should be noted that almost all the complications occurred in the subgroups of patients suffering from acute disease undergoing urgent procedure. This finding can be explained by the fact that in these patients there is already an ongoing infectious state which correlates with both surgical and post-procedural medical complications. Two recent meta-analysis of RCTs respectively comparing the four techniques for treating concomitant gallstones and CBD stones and iERCP + LC or preERCP + LC provided evidence that the combination of LC and iERCP appears to be the optimal strategy in terms of safety, technical success, and morbidity (13, 14).

Similar results come from a study comparing 18 studies regarding three management options (iERCP + LC, pre ERCP + LC, LCBDE). Laparoscopic cholecystectomy with an intraoperative endoscopic retrograde cholangiopancreatography is associated with the best overall outcomes (15).

From a therapeutic efficacy point of view, our study corroborates similar success rates and complications rates among the two different approaches, emphasizing the importance of offering patients a safe solution requiring only one OR access and cost reduction (16, 17).

The importance of iERCP in reducing postoperative bile leakage has been previously demonstrated (10). iERCP allows prompt detection and treatment of biliary complications, as evidenced by our series where one biliary leakage from the hepatic bed was successfully managed intraoperatively with a biliary stent placement. A recent retrospective study comparing LC + iERCP and LC + LCBDE concluded that the two techniques are comparable in terms of morbidity, mortality, technical success, mean hospital stay, readmission and reoperation rate, but iERCP is superior only for shorter operation time. The authors concluded that iERCP is preferred in centers with endoscopic expertise, while LCBDE remains suitable when endoscopic intervention is unavailable, or patients have complex anatomy making them unfit for ERCP (6).

As already reiterated by numerous retrospective analyses, the single stage approach has clear advantages compared to the two-stage approach. What the Covid period has added, however, is the awareness that the logistical problems that allow the single stage approach to be carried out can be overcome, and that this approach can be perpetuated continuously without excessive stress on the system.

This study has several limitations.

It is a retrospective study considering only cases fit for surgery.

The predominance of single stage cholecysto-choledocholitihiasis management reflects the prompt availability and skill of our endoscopic team. In most hospitals, two-stage approach remains more frequent due to logistic problems and lack of expertise. Despite this, the single stage approach was implemented during the covid pandemic, exhibiting good results without increasing complication rates. The single stage approach should therefore be taken into consideration for cholecysto-choledocolitiasis, as the coordination between suregons and endoscopist is achievable even in a complex scenario like covid pandemic.

As the care for these two patient groups was not provided during the same period and since a major pandemic was ongoing in the country where the study was performed for only one of the two patient groups, the differences in patients' outcomes could be in some way effected in unrelated ways to the COVID-19 pandemic. However, this same temporal separation minimized the selection bias, as patients did not choose between the two procedures but were assigned based on the period in which they required surgery, strengthening the results.

From the data we report it is clear that urgent cases are more challenging both from the endoscopic and the surgical point of view. It is therefore adivisable that such cases should be managed by experienced hands in order to minimize the complication rate.

However, according to our results and literature evidence, a shift toward a single stage definitive procedure for treating cholecysto-choledocholitiasis is advocated.

## Conclusions

Compared to preop ERCP + LC, LC + iERCP can reduce hospital stay, OR occupation time and hospital costs. The complication rate is not different between the two approaches.

The single stage approach should therefore be taken into consideration for cholecysto-choledocolitiasis unless there are insurmountable logistic issues or lack of endoscopic expertise to treat also challenging situations like urgent cases.

## Data Availability

The original contributions presented in the study are included in the article/Supplementary Material, further inquiries can be directed to the corresponding author.
